# Skeletal Muscle CLARITY: A Preliminary Study of Imaging The Three-Dimensional Architecture of Blood Vessels and Neurons

**DOI:** 10.22074/cellj.2018.5266

**Published:** 2018-03-18

**Authors:** Wenli Zhang, Shaohua Liu, Weichen Zhang, Wei Hu, Min Jiang, Amin Tamadon, Yi Feng

**Affiliations:** 1 Department of Digestive Diseases of Huashan Hospital, Fudan University, Shanghai, China; 2 Department of Sports Medicine, Huashan Hospital, Fudan University, Shanghai, China; 3 Department of Nephrology, Huashan Hospital, Fudan University, Shanghai, China; 4 State Key Laboratory of Medical Neurobiology and Institute of Brain Science, Brain Science Collaborative Innovation Center, Fudan University, Shanghai, China; 5 Department of Integrative Medicine and Neurobiology, School of Basic Medical Sciences, Shanghai Medical College, Institutes of Integrative Medicine of Fudan University, Shanghai, China

**Keywords:** CLARITY, Mouse, Muscle, Neuron, Vessel

## Abstract

**Objective:**

Passive CLARITY is a whole-tissue clearing protocol, based on sodium dodecyl sulfate (SDS) clearing, for imaging intact tissue containing transgenic or immunolabeled fluorescent proteins. In this study, we present an improved passive CLARITY protocol with efficient immunolabeling without the need for electrophoresis or complex instrumentation.

**Materials and Methods:**

In this experimental study, after perfusion of C57BL/6N mice with phosphate-buffered saline (PBS) and then with acrylamide-paraformaldehyde (PFA), the quadriceps femoris muscle was removed. The muscle samples were post-fixed and degassed to initiate polymerization. After removing the excess hydrogel around the muscle, lipids were washed out with the passive CLARITY technique. The transparent whole intact muscles were labeled for vessel and neuron markers, and then imaged by confocal microscopy. Three-dimensional images were reconstructed to present the muscle tissue architecture.

**Results:**

We established a simple clearing protocol using wild type mouse muscle and labeling of vasculatures and neurons. Imaging the fluorescent signal was achieved by protein fixation, adjusting the pH of the SDS solution and using an optimum temperature (37˚C) for tissue clearing, all of which contributed to the superiority of our protocol.

**Conclusion:**

We conclude that this passive CLARITY protocol can be successfully applied to three-dimensional cellular and whole muscle imaging in mice, and will facilitate structural analyses and connectomics of large assemblies of muscle cells, vessels and neurons in the context of three-dimensional systems.

## Introduction

Understanding the complex interactions between musclecells and other cell types found in vessels and neuronsis essential for delineating their roles in muscle functionand disease. Since most muscular diseases affect different muscle groups, the conventional method of skeletal muscleevaluation, which employs two-dimensional sectioningand imaging, does not provide a comprehensive picture ofcellular interactions between neighboring or distant cells inthe three-dimensional architecture of the muscle. Therefore, protocols need to be developed to simultaneously evaluatelarge populations of cells in muscles, such as blood, vascular and neuronal cells in three dimensions (1). 

Hitherto, several methods have been developed for the large-scale imaging of transparent and intact tissues with an emphasis on the central nervous system, including BABB (2), Scale (3), 3DISCO (4), ClearT (5), SeeDB (6), CLARITY (7), passive CLARITY (8), PACT (9), CUBIC (10, 11), FASTClear (12), SWITCH (13) and FACT (14). Among these approaches, hydrogel-based clearing protocols (including CLARITY, passive CLARITYand PACT) provide conditions for antibody labeling of tissue markers in animal models (7, 9). 

CLARITY uses electrophoretic tissue clearing to extractlipids from large samples faster than passive CLARITY andPACT, however, this results in the destruction of fine cellular structures (15) and initial attempts to use it in muscle tissueclearing did not provide satisfactory results (16). The PACT (9) and passive CLARITY (8) methods preserve the finetissue structure by avoiding electrophoretic tissue clearing, and using phosphate-buffered saline (PBS) (pH=7.5) andboric acid (pH=8.5) respectively as the solvent for sodiumdodecyl sulfate (SDS). Although the passive CLARITYprotocol has been used for clearing different tissues (Table 1), the clearing of muscle tissue with this method did not show antibody labeling (16, 17). 

**Table 1:** Successful applications of the passive CLARITY protocol for tissue clearing and three-dimensional imaging


Tissue/organ	Species	Hydrogel perfusion/embedding	Clearing solution	Clearing time	RI^*^ homogenization	References

Skeletal muscle (whole)	Mouse	+/+	4% SDS in boric acid (pH=8.5)	42 days (adult)	80% glycerol	Current study
Brain (whole)	Mouse	+/+	4% SDS in boric acid (pH=8.5)	21 days (adult)	FocusClear / 85-87% glycerol	(8)
Brain (section)	Mouse	+/+	4% SDS in boric acid (pH=8.5)	7 days (adult)	PBST	(18)
Brain (whole) / lung (whole) / testis (whole) / kidney (whole) / intestine (whole) / spleen (whole)	Mouse	+/+	4% SDS in boric acid (pH=8.5)	30 days (adult)	FocusClear / 80% glycerol	(19)
Brain (whole) / spinal cord (whole)	Mouse	+/+	4% SDS in boric acid (pH=8.5)	28-42 days (adult brain) / 14-28 days (adult spinal cord)	TDE	(20)
Brain (whole) / spinal cord (whole)	Mouse	+/+	4% SDS in boric acid (pH=7.5)	36 days (adult brain) / 21 days (adult spinal cord)	FocusClear	(21)
Brain (section) /spinal cord (section)	Mouse / rat	+/+	8% SDS in boric acid (pH=7.5)	4 days (adult mouse) / 6 days (adult rat)	80% Glycerol / 65% TDE	(22)
Brain (whole)	Rat	+/+	4% SDS in boric acid (pH=8.5)	28-56 days (adult)	RapiClear	(23)
Brain (section)	Rat	+/+	4% SDS in boric acid (pH=8.5)	6 days (age P0) to 20 days (age P24)	TDE	(24)
Brain (section)	Human	−/+	4% SDS in boric acid (pH=8.5)	14 days (adult)	ScaleA2 solution	(25)
Brain (whole)	Mouse / rat / human (section)	+/+	4% SDS in boric acid (pH=8.5)	21 days (adult mouse) / 60 days (adult rat) / 5-10 days (adult human)	87% glycerol / ScaleA2 solution	(26)
Cerebellum (whole)	Mouse / Human (section)	−/+	4% SDS in boric acid (pH=8.5)	7 days (adult mouse) / >28 days (human adult)	RIMS + PBS + Tween-20	(27)
Spinal cord (whole)	Mouse	+/+	4% SDS in boric acid (pH=7.5)	14 days (adult)	CUBIC clearing solution	(28)
Whole body	Zebrafish	-/+	8% SDS in boric acid (pH=8.5)	5-7 days (adult)	RIMS	(29)
Fetus (whole) / brain (whole) / lung (whole) / heart (whole) / kidney (whole) / muscle^†^ (whole)	Mouse	+/+	4% SDS in boric acid (pH=8.5)	3–10 days (fetus) / 10 days (other tissues)	RIMS	(17)
Liver (section)	Mouse	+/+	4% SDS in boric acid (pH=8.5)	30 days (adult)	RIMS	(30)
Lung (whole)	Mouse	−/+	8% SDS in boric acid (pH=8.5)	ND	RIMS	(29)
Intestine (section)	Mouse / human	+/+	4% SDS in boric acid (pH=8.5)	12–14 days (adult)	80% glycerol	(31)
Ovary (whole)	Mouse	+/+	4% SDS in boric acid (pH=8.5)	35 days (adult)	FocusClear	(32, 33)
Testis (whole)	Zebrafish	−/+	8% SDS in boric acid (pH=8.5)	13 days (adult)	RIMS	(34)
Stem-cell-derived cortical cultures	Mouse	ND	ND	ND	ND	(35)


*; ND; No data, PBS; Phosphate-buffered saline, PBST; Phosphate-buffered saline+Triton X-100, RI; Refractive index, RIMS; Refractive index matching solution, SDS; Sodium dodecyl sulfate, TDE; 2,20-thiodiethanol, and †; The passive CLARITY protocol was implemented on muscle tissue until the clearing stage (without immunolabeling and imaging).

Thus, it was necessary to develop a simple and improved method to clear thick muscle tissue by adjusting pH and temperature so as to preserve the cellular structure of muscle tissues. We modified the passive CLARITY method to achieve this goal. The hydrogel perfusion and embedding steps improved the preservation of proteins, and at 37°C and pH=8.5, protein loss was decreased and the proper conformation of the target proteins was maintained. The present study is thus the first to describe a simple improved passive CLARITY approach that provides optimal conditions for visualizing vessels and neurons in skeletal muscle.

## Materials and Methods

### Passive CLARITY of muscle 

In this experimental study, handling of animals and all experimental methods were conducted according to the Animal Research Ethic Guidelines of Fudan University, which conform to international guidelines. All procedures were approved by the Research Committee of Fudan University (Shanghai, China). Following a previously described protocol (32), C57BL/6N mice (Laboratory Animal LLC, China) were perfused transcardially while being alive with 40 ml ice-cold PBS solution (1 M, pH=7.6), followed by 20 ml of a mixture of 4% (w/v) paraformaldehyde (PFA), PBS (1 M, pH=7.6), 4% (w/v) acrylamide, 0.05% bis-acrylamide, 0.05% saponin (w/v) and 0.25% (w/v) VA-044 initiator in Millipore double-distilled water. The quadriceps femoris muscle was dissected and then post-fixed in the same perfusion solution at 4°C for three days.

**Fig.1:**
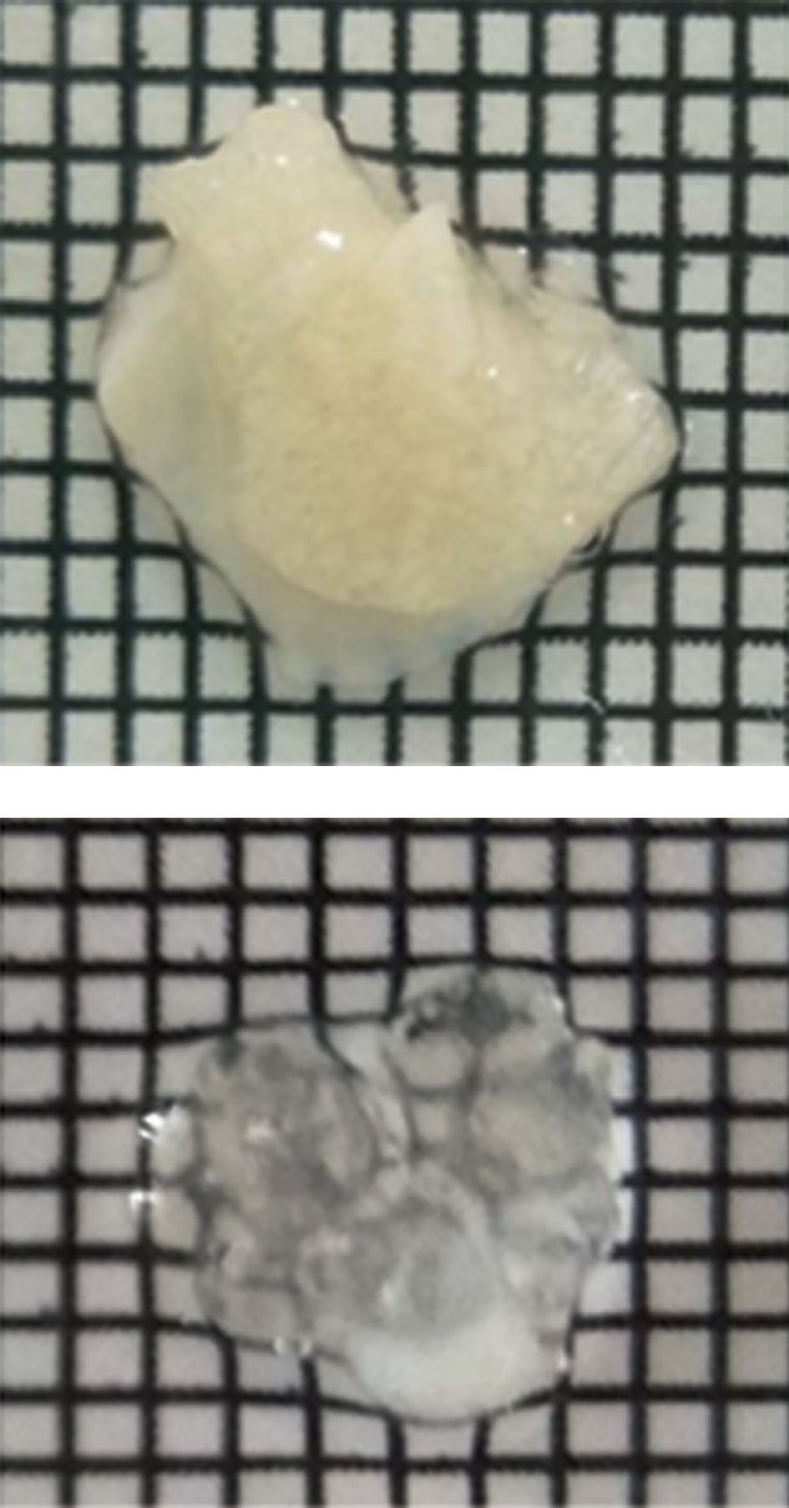
Transparency of mouse skeletal muscle before and after clearing with passive CLARITY.

The samples were then degassed by filling the tubes with fresh hydrogel monomer solution and incubated at 37°C (with shaking) to initiate polymerization of acrylamide. The excess hydrogel around the muscle was removed with tissue paper and lipids were washed out by passive clearing in a solution of 200 mM sodium borate buffer containing 4% (w/v) SDS (pH=8.5) at 37°C with gentle rotational shaking (15). The passive CLARITY solution was refreshed daily for three days and then changed weekly until complete transparency was reached. Before adding the fresh solution, its pH was checked and maintained at pH=8.5. The transparency of the tissue was checked on a daily basis using a graded paper (Fig .1). 

### Antibody staining and confocal imaging 

After clearing, the residual SDS was removed from the muscles by slow shaking in PBS with 0.1% Triton X-100 (PBST) for 24 hours. The samples were then incubated for three days with primary antibodies (Table 2) diluted in PBST. The samples were subsequently washed in PBST buffer for one day followed by incubation with secondary antibodies (Table 2) diluted in PBST for three days. To label cell nuclei, DAPI was added to the secondary antibody mixture for the final 12 hours of incubation. Before mounting and imaging, samples were washed in PBST for at least one day. All procedures were implemented with shaking at 37°C. 

The samples were embedded in a chamber formed by a flattened horse shoe-like piece of putty acting as a wall on a glass slide. The chamber was filled with 80% glycerol, and the upper part of the chamber was gently sealed using a Wellco dish [Pelco (Ted Pella), cat. no. 14032E120] with the glass surface facing down. This step prevented the formation of smalnl bubbles on the surface of the muscle. We used a Nikon A1R+ upright confocal microscope to obtain all confocal images presented here. 

**Table 2:** Details of antibodies used


Antibodies	Species	Dilution	Company	Cat. no	Markers for

Primary antibodies					
Tyrosine hydroxylase	Chicken	1:50	Abcam	ab76442	Neuron, muscle
CD31	Rabbit	1:10	Abcam	ab28364	Blood vessel
NeuN	Mouse	1:50	Abcam	ab104224	Neuron
Secondary antibodies					
Alexa Flour 488	Goat anti chicken	1:100	Life Technologies	A11039	
Alexa Flour 594	Goat anti rabbit	1:100	Life Technologies	A11012	
Alexa Flour 647	Goat anti mouse	1:100	Life Technologies	A-21235	
DAPI (4’,6-diamidino-2-phenylindole)		1:100	Life Technologies	D1306	Cell nucleus


**Fig.2:**
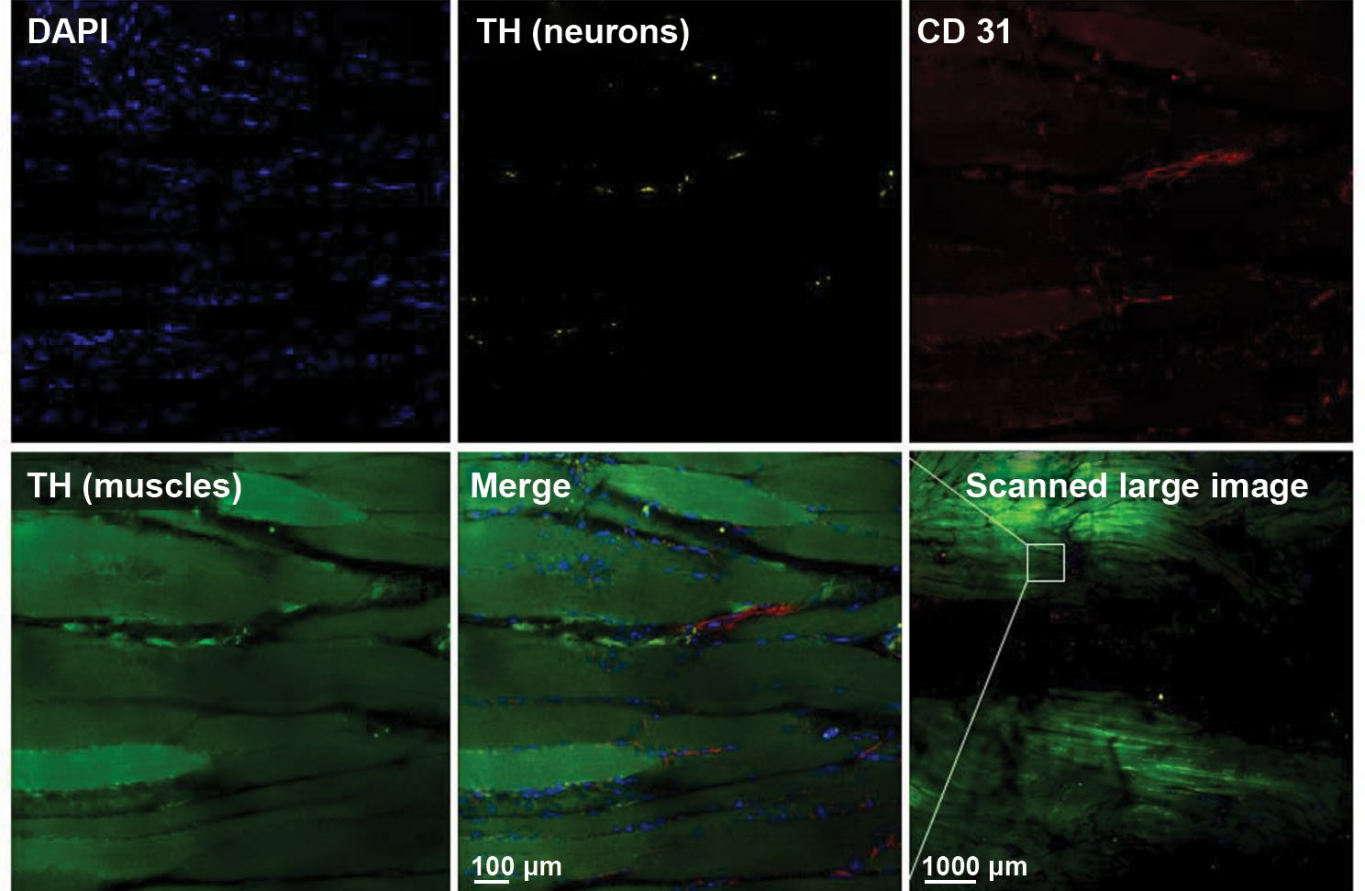
Immunostained mouse muscle cleared with the passive CLARITY protocol. Blood vessels (CD31), neurons (tyrosine hydroxylase, after removing background with the “background subtraction option” of Imaris), muscle bundles (tyrosine hydroxylase) and cell nuclei (DAPI) have been labeled. The tissue was scanned with the large image scan option using confocal microscopy at ×25 magnification. The passive CLARITY method also immunostained vessels, neurons and their nuclei in the tendon of the quadriceps femoris (the central black part of the image).

**Fig.3:**
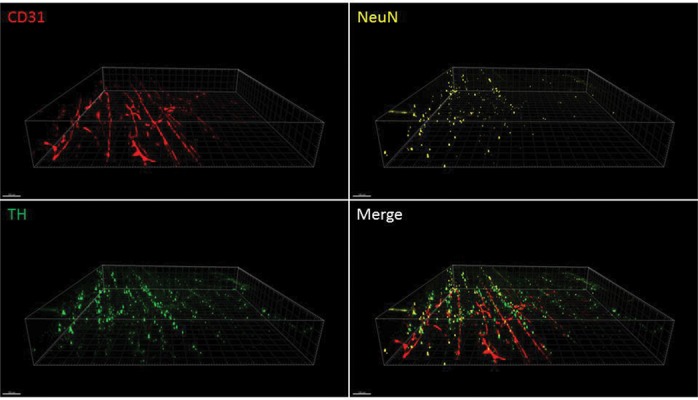
High-resolution imaging of mouse muscle cleared with the passive CLARITY protocol. Blood vessels (CD31) and neurons (tyrosine hydroxylase and NeuN) are labeled. Confocal microscopy was used at ×25 magnification with an area of 1024×1024 µ and Z of 250 µm.

After fixing the embedded apparatus on the microscope stage, we used a water immersion ×25 objective lens to focus the laser onto the specimen (1.1-NA, 2 mm-WD, Nikon, USA), and the muscle tissue was scanned using the large-image scan option of the microscope. Prior to Z-scanning, the laser power, light gain and offset of the upper and lower visible surfaces of the scanning slice were defined for maximum acquisition of excitation and emission of different secondary antibody signals using the intensity correction option of the Nikon NIS software. After selecting the appropriate field on the scanned large image, the objective lens was placed on the upper layer of the muscle, and three fields (XY=1024×1024 µ) with whole tissue depth (Z=maximum visible signals down to 250 µm) were scanned (speed=0.5, step distance=1 µm). After obtaining the images, TIFF image sequences were transferred to Bitplane Imaris software (version 7.4.2) for Z-stack image acquisition and three-dimensional reconstruction. 

### Three dimensional reconstruction 

The three dimensional (3D) reconstruction and tracing of vessel and neuron morphology was undertaken using Imaris and its tools, including Surface and Filament, and automatic or semiautomatic signal detection. Because of the large amount of data, a workstation server was used for data analysis with the specifications of Dell server board T7910, two Intel E5-2687WV4 CPUs, four ~32 GB DDR4 ECC RAM, a ~4 TB hard disk (Dell SAS 7.2K), and an NVidia Quadro 5000 graphics card. Finally, for the second round of labeling with new antibodies, the muscle tissue was incubated in the clearing solution for 24 hours at 37°C on the shaker. After washing with PBST, the same protocol of immunostaining was then undertaken with new antibodies.

## Results

Using this improved passive CLARITY method for skeletal muscle, we were able to specifically stain vessels, neurons and nuclei (Figes.2, 3) and non-specifically stain muscle bundles by tyrosine hydroxylase (Fig .2). In addition, as shown in this figure the passive CLARITY method also immunostained vessels, neurons and their nuclei in the tendon of the quadriceps femoris. This finding demonstrates that passive CLARITY can be used not only for clearing but also three-dimensional imaging of tendons despite the structural rigidity and mostly fibrous composition of the tendon in comparison with muscle composition.

## Discussion

In this study, we have made a number of modifications to the passive CLARITY method on brain tissue (8) to successfully image mouse muscle. Milgroom and Ralston (16) reported that CLARITY clears hind limb skeletal muscles in mice but does not allow labeling of target molecules with fluorescent markers, however, this improved method is a simple technique that enables muscle tissue imaging. The mice in this study were not perfused with hydrogel before post-fixing the muscles in the hydrogel solution. The recently described fast free of-acrylamide clearing tissue (FACT) protocol showed that hydrogel can be removed from the fixative solution in brain tissue samples during SDS whole tissue clearing (14), however, exposing the tissue to electrophoresis at high temperature (50°C), as reported by Milgroom and Ralston (16), may increase protein loss. In our modified protocol, we used cold hydrogel perfusion and a passive method of clearing at 37°C, resulting in reduced protein loss in the muscle samples. 

In addition to optimizing the temperature, controlling pH during the clearing process is a key factor for increasing the efficiency of passive CLARITY of muscle tissue. In our protocol, maintaining the pH at 8.5 resulted in appropriate labeling. While not addressed in the previous, unsuccessful study (16), pH fluctuation of the clearing solution during electrophoresis may have caused increased protein structure deformity (31), given that changes in pH occur faster during electrophoresis than in passive CLARITY (36). In addition, controlling the clearing time and assessing the transparency of the sample during clearing will reduce protein loss. Although the clearing time of the analyzed muscles (soleus, extensor digitorum longus and flexor digitorum brevis) was not provided in Milgroom and Ralston’s study (16), it should be shorter than our protocol considering the volume of the muscle and the clearing protocol. In passive CLARITY, the clearing duration was 40 days for whole mouse quadriceps femoris. In addition, in our protocol, matching the refractive index (37) of muscle tissue after clearing increased the depth of access to fluorescent signals to 250 µm, which is 2.5-fold deeper than the reported depth (97 µm) by Milgroom and Ralston (16).

## Conclusion

Successful labeling of vessels, neurons and nuclei in skeletal muscle, after clearing by the improved passive CLARITY approach, resulted in 3D imaging of their architecture in skeletal muscle for the first time. Although in the previous, unsuccessful method details of the antibodies or method of staining were not mentioned and the authors only reported that actin and α-bungarotoxin labeling was unsuccessful, it seems that the three main reasons for their lack of success may be related to i. The characteristics of the antibodies (in the passive CLARITY technique, C-terminal primary antibodies are better for staining than N-terminal antibodies), ii. The method of staining, and iii. The amount of protein loss in samples of which the latter could be determined by measuring the amount of protein in the clearing solution. Finally, the passive CLARITY protocol developed here permits multiple rounds of staining of the muscle with different antibodies.
